# Preparation of Crude Subcellular Fractions by Differential Centrifugation

**DOI:** 10.1100/tsw.2002.851

**Published:** 2002-06-13

**Authors:** John Graham

**Affiliations:** School of Biomolecular Sciences, Liverpool John Moores University, UK

**Keywords:** homogenate, rat liver, differential centrifugation, nuclear pellet, heavy mitochondrial pellet, light mitochondrial pellet, microsomes, cytosol

## Abstract

The employment of differential centrifugation to prepare crude fractions of subcellular particles from homogenates is often a necessary first step to a subsequent purification of one or more particles on a density gradient. Buoyant density gradient purification of peroxisomes or lysosomes for example is almost invariably carried out on a light mitochondrial fraction so as to eliminate smaller particles that may have similar densities. Unless they are first removed, large rapidly sedimenting particles in homogenates may also disturb shallow gradients designed to fractionate small low-density microsomes.

**METHOD TYPE:** extraction, isolation, purification and separation

**SUB METHOD TYPE:** centrifugation

## INTRODUCTION

This Protocol Article describes the use of differential centrifugation to fractionate a mammalian liver homogenate but similar methods should be applicable to all mammalian tissues, cultured cells, and organisms such as yeast, as long as an efficient homogenization procedure is available. Refs. [1,2,3] describe many of these procedures in more detail.

## MATERIALS AND EQUIPMENT

The solutions used for homogenization and for washing and resuspension of the pellets, depends upon the organelle to be purified. They were developed for work with rat liver and other soft tissues and generally contain sucrose as the osmotic balancer. Many cultured cells can also be homogenized in the General Purpose medium or some other similar isoosmotic medium[4]. If, however, the homogenization has been carried out in a hypo-osmotic medium, then this should be adjusted to the recommended concentration of sucrose and other additives as soon as possible.

**General Purpose**: 0.25 M sucrose, 1 mM EDTA, 10 mM Hepes-NaOH, pH 7.4
**Nuclei**: As General Purpose but replace 1 mM EDTA with 25 mM KCl, 5 mM $MgCl_2$
**Peroxisomes**: Add 0.1% ethanol to General Purpose
**Mitochondria**: 0.2 M mannitol, 50 mM sucrose, 1 mM EDTA, 10 mM HEPES-NaOH, pH 7.4

Low-speed refrigerated centrifuge with swinging-bucket rotor to hold 30- to 40-ml polycarbonate tubes (see Notes 1 and 2).
High-speed centrifuge with fixed-angle rotor to hold 30- to 40-ml polycarbonate tubes (see Notes 1 and 2)
Ultracentrifuge with fixed-angle rotor to hold 30- to 40-ml polycarbonate tubes (see Notes 1 and 2)
Dounce homogenizer (loose-fitting, Wheaton Type B)

## METHOD

All operations should be carried out at 0–4°C and all solutions should be precooled on ice**.**

1. Prepare the homogenate according to one of the strategies given[5,6].
2. If the nuclear pellet is to be processed, filter the homogenate through four layers of cheesecloth or fine nylon mesh (pore size 75 µm) to remove any unbroken cells and connective tissue. This filtration is not normally necessary for cultured cells.
3. Pellet the **nuclear fraction** by centrifugation at 1,000$g_{av}$ for 10 min (see step 7 and Notes 3–5).
4. Pellet the **heavy mitochondrial fraction** by centrifuging the postnuclear supernatant at 3,000$g_{av}$ for 10 min (see step 7 and Notes 3–5).
5. Pellet the **light mitochondrial fraction** by centrifugation of the heavy mitochondrial supernatant at 15,000–17,000$g_{av}$ for 10 min (see step 7 and Notes 3–5).
6. Pellet the **microsomal fraction** by centrifuging the light mitochondrial supernatant at 100,000$g_{av}$ for 45 min (see Notes 3–5).
7. Resuspend all pellets in the appropriate medium by gentle homogenization with a loose-fitting Dounce homogenizer (approx. 0.5-mm clearance) to ensure complete dispersion of the pellets.

## NOTES

1. Centrifugation of the nuclear pellet is very often carried out in a swinging-bucket rotor (low-speed centrifuge) and all the subsequent steps in a fixed-angle rotor. To achieve the best resolution however, a fixed-angle rotor should be used for all differential centrifugation. The shorter the sedimentation path length of the rotor, the better will be the resolution. Swinging-bucket rotors tend to have long path lengths and the only merit of their use is that the pellet, being formed at the bottom of the tube, is rather more stable and less likely to slide during decantation. In fixed-angle rotors always decant "away" from the pellet. In the case of the 1000*g* pellet however the nuclei and debris are so large and rapidly sedimenting, compared to the other particles, that the long path length of a swinging-bucket rotor is not a real disadvantage.

2. The rotor tube volume can be scaled up or down as required, the volumes given are typical for a single rat liver (approx. 10 g).
3. To improve the recovery of more slowly sedimenting particles and increase the purity of the differential centrifugation fractions it may be necessary to wash the pellets, in which case the resuspended pellets should be adjusted to about half of the volume of the homogenate and then recentrifuged at the same speed and time. The two supernatants are then combined prior to centrifugation at the next step. Sometimes this washing is extended to three or more cycles of resuspension and recentrifugation; e.g., for the purification of mitochondria from the 3000*g* pellet. Although the washing procedure can produce gains in recovery and purity of particles: it should always be a primary aim to minimize the amount of pelleting and resuspending as this causes progressive fragmentation of particles. It is also very time consuming.
4. **Composition of the pellets -** The composition of the various fractions produced by differential centrifugation have been well defined for commonly used tissues such as mammalian liver, but for many cultured cells the distribution of the various membrane particles is rather less clear. The **Nuclear Pellet** contains, in addition to nuclei, mitochondria, sheets of plasma membrane (if present) and, if the homogenate has not been filtered, unbroken cells and debris (including connective tissue). Formation of this pellet is sometimes carried out at 500*g* rather than 1000*g*. The **Heavy Mitochondrial Pellet** contains, predominantly, mitochondria with rather few contaminants and is a common source of these organelles for respiratory studies. Minor components such as lysosomes, peroxisomes, Golgi membranes, and various membrane vesicles are present largely because of entrapment during the pelleting process. Some plasma membrane fragments may also be present. These contaminants can be reduced by repeated washing. The **Light Mitochondrial Pellet** contains mitochondria, lysosomes, peroxisomes, Golgi membranes, and some endoplasmic reticulum. Of all differential centrifugation fractions it is the most variable in terms of the actual centrifugation parameters used: g-forces of 15,000–20,000*g* and times of 10–20 min are the most common. Some methods are designed to maintain the Golgi membranes in their "stacked" form so that they sediment at much lower g-forces (see [2] for more information). The **Microsomal Pellet** is rather better defined and contains only membrane vesicles. Some of those vesicles will have been present in the cell before homogenization (e.g., endosomes, secretory vesicles, and vesicles from the *trans*-Golgi network), others from the plasma membrane, Golgi, and smooth and rough endoplasmic reticulum, will have been produced by the homogenization procedure. Ref. [3] discusses differential centrifugation in more detail.
5. **Analysis of the pellets -** Although the operator may be interested only in processing one of the pellets, it is nevertheless important to analyze all of the pellets for chemical and enzyme markers. This will allow determination of the recovery, not only of the particle of interest but also of contaminants that may be difficult to remove. Analysis of the cytosolic fraction (100,000*g* supernatant) should always be included; this not only permits complete and valuable “book-keeping” of organelle markers, it can also give information on possible disruption to organelles and consequent release of organelle

**TABLE 1**
**Chemical and Enzyme Markers for Subcellular Membranes**

| Subcellular Particle | Marker* | Comment |
|---|---|---|
| Nucleus | DNA | |
| Mitochondria | Succinate dehydrogenase | Glutamate dehydrogenase is another commonly used marker |
| Lysosomes | Acid phosphatase, β-galactosidase | Other hydrolytic enzymes, e.g., β-*N*-acetylglucos- aminidase, may be used |
| Peroxisomes | Catalase | Method using titanium oxysulphate much easier to execute than one using $KMnO_4$ |
| Endoplasmic reticulum (ER) | NADPH-cytochrome c reductase, rotenone-insensitive NADH-cytochrome c reductase | Glucose-6-phosphatase is only a reliable marker for liver and kidney ER. |
| Rough ER | As ER plus RNA | |
| Golgi | UDP-galactose galactosyl transferase | Method using ovalbumin as galactose acceptor is easiest to execute. Properly a marker only for the *trans*-Golgi. |
| Plasma membrane | 5'-nucleotidase, $Na^+/K^+$-ATPase, leucine aminopeptidase, alkaline phosphatase | Only 5'-nucleotidase and alkaline phosphatase are reliable markers for cultured cells. Plasma membrane domain specificity of these markers is common in morphologically polarized cells. |

* For more information see [7,8].

contents during the homogenization procedure. In addition to protein, appropriate markers (Table 1) should be measured.

## ACKNOWLEDGEMENTS

The author and TheScientificWorld wish to thank Axis-Shield PoC, AS, Oslo, Norway for their kind permission to adapt OptiPrep™ Application Sheet S7 in the preparation of this Protocol Article.
